# Abusive head trauma in court: a multi-center study on criminal proceedings in Germany

**DOI:** 10.1007/s00414-020-02435-5

**Published:** 2020-10-08

**Authors:** Katharina Feld, Dustin Feld, Bernd Karger, Janine Helmus, Nneka Schwimmer-Okike, Heidi Pfeiffer, Sibylle Banaschak, Daniel Wittschieber

**Affiliations:** 1 Institute of Legal Medicine, University Hospital Cologne, Cologne, Germany; 2 adiutaByte GmbH, Business Campus, Sankt Augustin, Germany; 3 Institute of Legal Medicine, University Hospital Münster, Münster, Germany; 4 Institute of Legal Medicine, University Hospital Essen, Essen, Germany; 5 Institute of Legal Medicine, Jena University Hospital, Friedrich Schiller University, Am Klinikum 1, 07747 Jena, Germany

**Keywords:** Child abuse, Clinical forensic medicine, Perpetrator’s confessions, Perpetrator’s profile, Legal assessment

## Abstract

The shaken baby syndrome (SBS) is a common variant of abusive head trauma (AHT) in infants and toddlers. Data on the legal outcome of such cases are still sparse. By means of a retrospective multi-center analysis, 72 cases of living children diagnosed with SBS/AHT from three German university institutes of legal medicine were identified. Forty-six of these cases with 68 accused individuals were available and could be evaluated with regard to basic data on the course of the criminal proceedings as well as the profile of the defendants (sub-divided into suspects, convicts, and confessed perpetrators). Criminal proceedings predominantly commenced with a complaint by the treating hospital (62%) and were found to be closed (without judgment) in 50% of the cases, mostly due to a “lack of sufficient suspicion.” Of the 23 cases with judgment, the court decided on acquittal in 4 cases (17%). Imprisonment was the most frequent sentence (16 out of 19 cases with conviction, 84%), whereby the sentence has been suspended on probation in 63% of the cases. Suspects and perpetrators were mostly male and derived from the close family environment of the injured children. All confessed perpetrators stated an “excessive demand” as the reason for the violent shaking of the child. The results of the present study are in line with data from other studies with other legal systems. As many criminal proceedings were closed and the 4 acquittals occurred because the perpetration could not be ascribed to a specific perpetrator, improving the forensic methods for such an unequivocal assignment would be desirable.

## Introduction

Abusive head trauma (AHT) is predominantly encountered in infants and toddlers within their first 2 years of life [1, 2]. The incidence of AHT in the western world ranges between 20 and 40 per 100,000 children under the age of 1 year [1–7]. While the proportion of survivors with long-term damage is reported to be between 62 and 96%, lethality is described with 13–36% [8].

A common variant of AHT is the so-called shaken baby syndrome (SBS), which is still diagnosed explicitly, at least in many European countries. The American Academy of Pediatrics (AAP) recommends the term AHT for diagnostics and medical communication, arguing that its etiology is multifactorial and SBS represents one possible injury mechanism only [9]. As pathophysiological and biomechanical mechanisms of the SBS are not yet fully understood, discussions and controversies on these aspects are still present, especially in court [10, 11]. In 2018, a consensus statement on AHT, supported by numerous European and American medical societies, was published to clarify the diagnostic workup, to reduce confusions, and to counter speculative theories frequently brought up in court [12].

Crying is discussed as a typical trigger for the violent shaking of a child [13–15]. The perpetrator has most frequently been identified as the father, the mother’s new partner, the mother, or the female babysitter [16]. Excessive demand in combination with low frustration tolerance and a lack of impulse control were stated as reasons for the violent act [15].

Inherently, given the high injury potential with possibly lethal outcome, suspects frequently become subjects of criminal proceedings. Only limited data are available concerning the course and outcome of these trials [17–20]. Especially, data from European legal systems are sparse, and data from Germany are missing completely. However, such data may help identify more promising starting points for prevention programs. Forensic physicians who are commissioned by authorities to answer the question of whether AHT has occurred or not may also benefit from a better understanding of the requirements at trials of AHT cases.

Therefore, the present study aimed at investigating the criminal proceedings of AHT cases of a 10-year period at German criminal courts, including analyses of the profiles of the defendants. The results will finally be compared with data from other legal systems.

## Material and methods

### Ethical approval

The ethical boards of all participating institutions, which are the university hospitals of the cities of Cologne, Essen, and Münster, approved the present study, reference number: 2014-658-f-N (ethics committee of the Medical Association of Westfalen-Lippe and the Westphalian Wilhelms University).

### Collection of cases

The medico-legal expert opinions on individuals living at the time of examination and carried out between 2006 and 2015 at the University Institutes of Legal Medicine of the German cities of Cologne, Essen, and Münster were retrospectively reviewed as to the diagnosis of “shaken baby syndrome,” which is the most common diagnosis of AHT cases in Germany. Such expert opinions represent the best reference standard for diagnosing and evaluating AHT cases in Germany and are usually based on a medico-legal physical examination, the complete clinical records, and the first criminal investigation results by the police. Of 72 identified cases, the completed criminal investigation files of the 13 departments of public prosecution responsible for these cases were accessed after approval and provision. In total, 46 case files were available for analysis.

### Data acquisition

The criminal investigation files of the collected cases were analyzed by two specialists with board certification in forensic medicine (KF and DW), both of them with extensive experience in medico-legal assessment of SBS/AHT cases. All key facts as well as all demographic and forensically relevant data on both the criminal proceedings and the defendants were recorded. The latter were subdivided into suspects, convicts, and confessed perpetrators.

### Statistical analysis

All statistical evaluations were performed using the programming language and free software environment for statistical computing “R” (version 3.6.1). In detail, the basic statistical functions like mean(x, …) and median(x, na.rm = FALSE, ...) have been applied. The binomial {stats} functions have been used to estimate the *p* values, e.g., dbinom(x, size, prob., log = FALSE). Statistical significance has been established from *p* < 0.05.

## Results

Table 1 shows the general characteristics of the criminal proceedings investigated in this study. In total, 46 criminal proceedings including 68 accused individuals were available for further analysis. The mean duration of criminal proceedings was 15.5 months (range 1–56 months). In the majority of the cases (*n* = 33, 72%), the offense was initially indicated by the treating hospital the child had been admitted to. After further police investigations, the responsible Department of Public Prosecution pressed charges against suspects in 59% of the cases (*n* = 27). In half of the total cases (*n* = 23, 50%), the criminal proceedings were closed (without judgment), mostly due to “lack of sufficient suspicion.” In the other half of the cases with court judgment, the persons charged were acquitted in 4 out of 23 cases (17%). Imprisonment was the most frequent sentence (16 out of 19 cases with conviction, 84%). Prison sentences ranged from 3 months to 6 years, community work sentences from 50 to 150 h, and in 1 case the fine was € 1000. In 12 out of 19 cases with conviction (63%), the sentence was suspended on probation.

**Table 1:** General characteristics of the criminal proceedings

	Value(s)
Number of criminal proceedings	46
Number of individuals accused	68
Total duration in months^1^ (mean; median; range)	15.5; 13.5; 1–56
Complainant, *n* (%)^2^	
Treating hospital (pediatric ward)	33/46 (72)
Mother of the child	6/46 (13)
Youth welfare office	5/46 (11)
Department of Forensic Medicine	4/46 (9)
Father of the child	3/46 (7)
Aunt of the child	1/46 (2)
Lawyer of the child	1/46 (2)
Charges brought against suspect(s), *n* (%)	
Yes	27/46 (59)
No	19/46 (41)
Final result of the criminal proceeding, *n* (%)^3^	
Closing of the proceedings (without judgment)	23/46 (50)
*Due to lack of sufficient suspicion*	15/23 (65)
*Due to missing attribution of the offense to an offender*	8/23 (35)
*Due to insufficient reason*	3/23 (13)
Court judgment	23/46 (50)
*Conviction*	19/23 (83)
*Acquittal*	4/23 (17)
Type of sentence, *n* (%)^4^	
Imprisonment	16/19 (84)
Community work	3/19 (13)
Fine	1/19 (5)
Suspended sentence (probation), *n* (%)	
Yes	12/19 (63)
No	7/19 (37)

^1^From the date of the criminal complaint to the date of the termination of the proceeding

^2^Numbers do not add up to *n* = 46 (100%) due to multiple complainants in some cases

^3^Numbers do not add up to *n* = 23 (100%) due to multiple justifications for dismissal of the proceeding

^4^Numbers do not add up to *n* = 19 (100%) because 1 individual was sentenced to both community work and fine

*n* = number of cases

% = percent

**Fig. 1:**
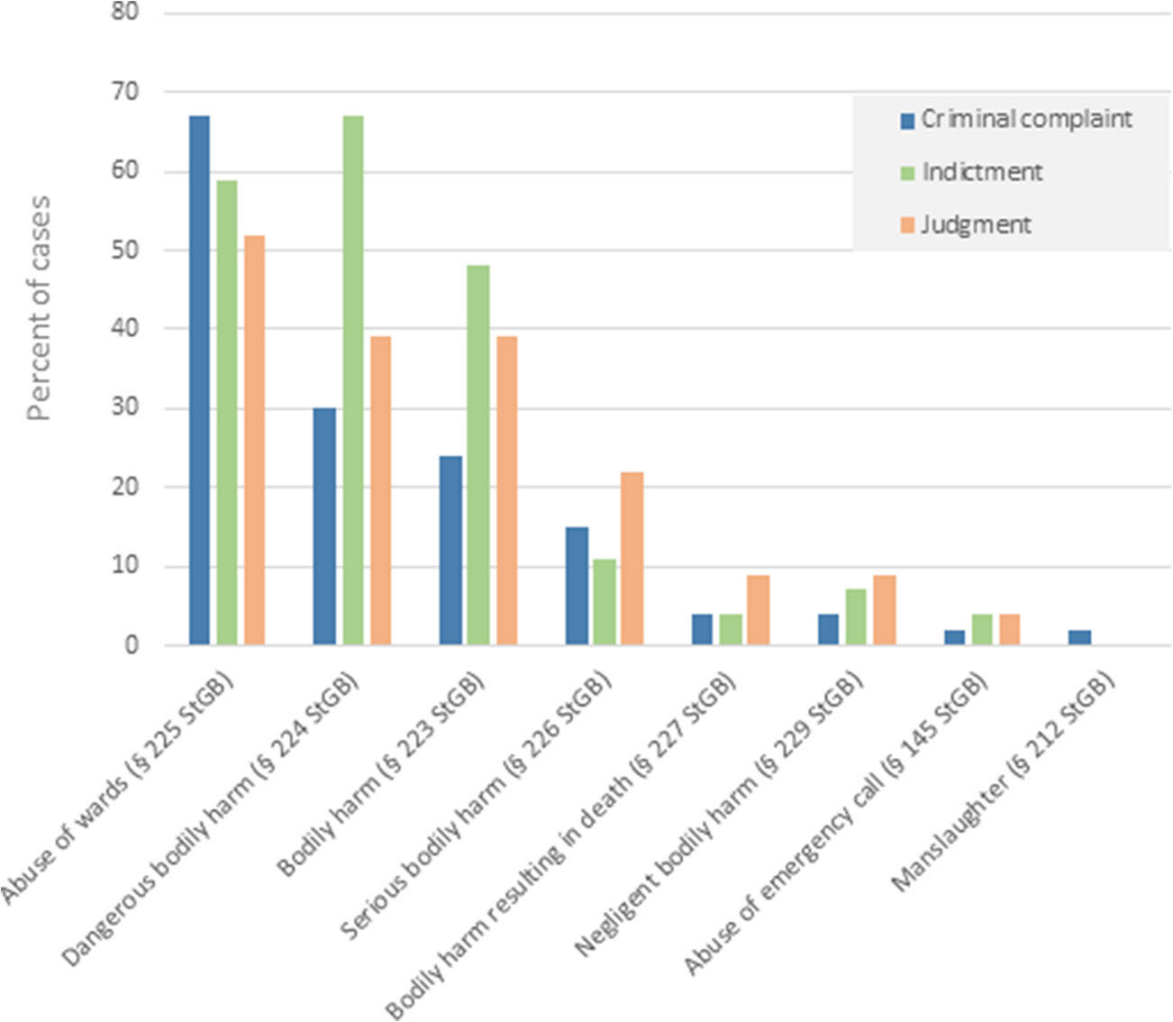
Criminal offenses charged according to criminal complaint, indictment, and judgment, whenever available**.** StGB is the German abbreviation for “Strafgesetzbuch” (= German Criminal Code)

Figure 1 shows the frequencies of the charged criminal offenses (according to StGB—Strafgesetzbuch = German Criminal Code) found in criminal complaint, indictment, and judgment, whenever available. “Abuse of wards” (§ 225 StGB) represents the most frequent criminal offense in both the criminal complaint (*n* = 31, 67%) and the judgment (*n* = 12, 52%). However, “dangerous bodily harm” (§ 224 StGB) was the most frequent criminal offense charged within the indictments (*n* = 18, 67%). Death occurred afterwards in 3 cases.

**Fig. 2:**
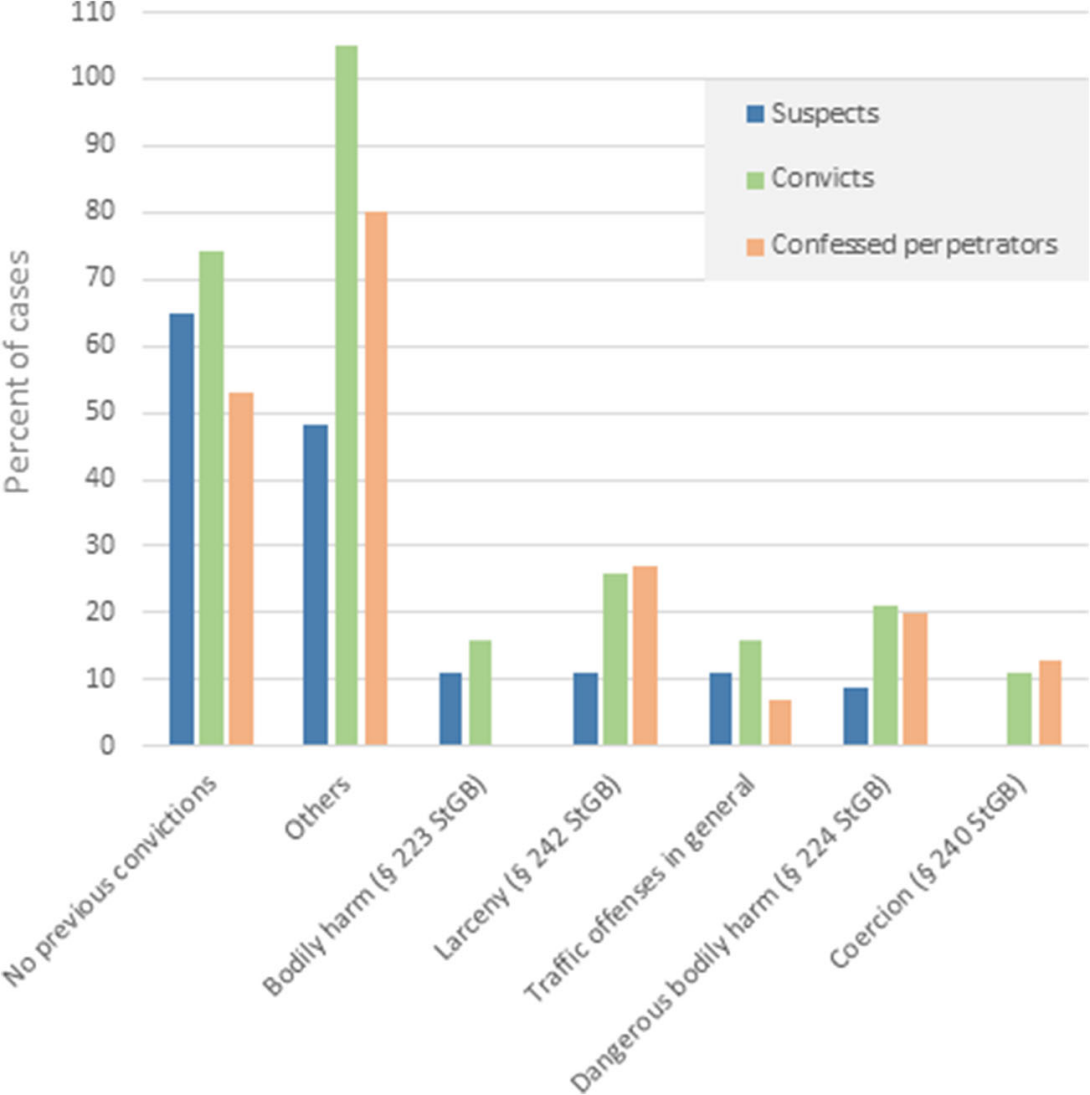
Previous convictions in suspects, convicts, and confessed perpetrators**.** “Others” do not add up to 100% due to the multiple offenses per case; these include abuse of wards, resistance against law enforcement officers, fraudulent acquisition of services, damage of property, robbery, burglary, insult, false statement, illegal possession of arms, fraud, false reason for suspicion, abuse of emergency calls, sexual abuse of children, threat, fencing, and embezzlement. StGB is the German abbreviation for “Strafgesetzbuch” (= German Criminal Code)

Table 2 presents a comparison of the profiles of suspects, convicts, and confessed perpetrators. From the 68 suspects of the 46 case files, 21 individuals (31%) in 19 cases were convicted by court. In two of these cases, both mother and father of the child were convicted. Sixteen individuals (24%) in 15 cases confessed to violent shaking of the child, whereof 14 were convicted, while 2 criminal proceedings were closed without judgment. With respect to sex, male individuals—the father of the child in particular—dominate in all three groups. No statistical significance could be achieved concerning the distribution of the sexes within the group of suspects (*p = 0.19*), whereas the difference between males and females within the group of confessed perpetrators was statistically significant (*p = 0.04*). Figure 2 additionally shows the previous convictions, differentiated by these 3 groups.

**Table 2:** Comparison of the profiles of suspects, convicts, and confessed perpetrators

	Suspects	Convicts	Confessed perpetrators
Number of case files, *n* (%)	46 (100)	19/46 (41)	15/46 (33)
Number of individuals, *n* (%)	68 (100)	21/68 (31)	16/68 (24)
Age in years (mean; median; range)	27.7; 25; 15–60	25.1; 24; 18–39	23.0; 23; 18–30
Sex, *n* (%)			
Male	38/68 (56)	12/21 (57)	12/16 (75)
Female	30/68 (44)	9/21 (43)	4/16 (25)
Relationship to victim, *n* (%)			
Father	34/68 (50)	11/21 (52)	12/16 (75)
Mother	26/68 (38)	9/21 (43)	4/16 (25)
New partner of the mother	2/68 (3)	1/21 (5)	–
Grandmother	2/68 (3)	–	–
Aunt	2/68 (3)	–	–
Grandfather	1/68 (1)	–	–
No familial relationship	1/68 (1)	–	–
Nationality, *n* (%)^1^			
German	61/68 (90)	20/21 (95)	14/16 (88)
Iraqi	2/68 (3)	–	–
Turkish	2/68 (3)	–	–
Russian	2/68 (3)	1/21 (5)	2/16 (13)
Portuguese	1/68 (1)	–	–
Belgian	1/68 (1)	1/21 (5)	1/16 (6)

^1^Numbers do not add up to 100% due to dual citizenship in one individual

*n* = number of cases

% = percent

Table 3 shows the analysis of statements made by the suspects. In approximately two-thirds of the cases (*n* = 31), the suspect made a statement on the origin of the child’s injuries, whereof 15 cases (48%) contained a confession of violent shaking of the child. In 9 of these cases (60%), a confession was made as early as prior to the trial (2 of them by the defense lawyer) and in 6 cases (40%) as late as in the course of the trial. Two confessions of violent shaking included the statement of the presence of a so-called brief resolved unexplained event (BRUE, according to [21]) prior to shaking of the baby. In all cases with confession, “excessive demand” was stated as the reason for violent shaking. Among the cases with statements containing alternative explanations for the injuries of the child, a fall from low height (e.g., from dining table, bathtub, or sofa) was the most frequent statement (*n* = 8, 44%). Stating a BRUE did not occur in conjunction with alternative explanations for the injuries of the child, especially not when “non-violent shaking for resuscitation” was indicated. Two suspects first stated an alternative explanation for the injuries of the child (a. non-violent shaking for resuscitation, b. tossing of the child in the air with subsequent catching) but later confessed to violent shaking (a. violent shaking + BRUE stated prior to shaking, b. violent shaking without restrictions).

**Table 3:** Statements made by suspects (*n* = 46; 100%)

	*n* (%)
Statement given^1^	31/46 (67)
Statement contains a confession of violent shaking of the child	15/31 (48)
Statement contains alternative explanations for the injuries of the child	18/31 (58)
No statement given	15/46 (33)
Statement contains a confession of violent shaking of the child	
Confession of violent shaking (without restrictions)	13/15 (87)
Confession of violent shaking + BRUE stated prior to shaking	2/15 (13)
Excessive demand given as reason ^2^	15/15 (100)
Calming given as reason ^2^	2/15 (13)
Statement contains alternative explanations for the injuries of the child^3^	
Fall from low height	8/18 (44)
Non-violent shaking for resuscitation (without claimed BRUE prior to shaking)	3/18 (17)
Slight and non-violent shaking for waking up	2/18 (11)
Tossing of the child in the air with subsequent catching	3/18 (17)
Tossing and falling	1/18 (6)
Sickness with vomiting	1/18 (6)
Injury caused by siblings (standing on head, neck, and upper body)	1/18 (6)
Vaccination	1/18 (6)
Playful jumping and dancing by father	1/18 (6)
Ride in the pram	1/18 (6)
Obstetrical injury	1/18 (6)

^1^Numbers do not add up to *n* = 31 (100%) due to multiple statements in some cases

^2^Numbers do not add up to *n* = 15 (100%) due to multiple reasons stated in some cases

^3^Numbers do not add up to *n* = 18 (100%) due to multiple and/or changing statements in some cases

*n* = number of cases

% = percent

BRUE = Brief resolved unexplained event

## Discussion

Using data from three German university institutes of legal medicine, this retrospective multi-center study on 46 SBS/AHT cases with 68 accused individuals evaluated basic data on the course and the outcome of the criminal proceedings as well as the profile of the defendants. To this day, studies with a comparable research approach, i.e., including information on defendants, confessions, or convictions, are sparse and were predominantly published in pediatric journals [17–20, 22–25] (Table 4). In these studies, there are various thematic focuses, such as the relationship between suspects and victims [17] and the correlation of confessed AHT cases with neurological or radiological findings [22–25]. In 5 of the comparative studies, information on criminal law aspects is also available [18–20, 22, 24]. In view of the scarcity of available studies, a structured and detailed analysis of criminal proceedings, as addressed in the present study, appears all the more important.

**Table 4:** General characteristics of studies investigating the outcome of criminal proceedings in cases of SBS/AHT

Study	Origin	Study period (years)	Number of SBS/AHT cases	Cases with identified suspects, *n* (%)^1^	Cases with confessed perpetrators, *n* (%)	Cases with convictions, *n* (%)
Starling et al. (1995)	USA	12	151	127/151 (84)	37/151 (25)	31/151 (21)
Ricci et al. (2003)	USA	4	19	15/19 (79)	4/19 (21)	9/19 (47)
Starling et al. (2004)	USA	21	171	171/171 (100)	81/171 (47)	n/a
Biron et al. (2005)	Australia	10	52	n/a (52?)	5/52 (10)	n/a
Kelly et al. (2009)	New Zealand	11 (19)	39	20/39 (51)	19/39 (49)	14/39 (36)
Adamsbaum et al. (2010)	France	7	112	112/112 (100)	29/112 (26)	n/a
Esernio-Jenssen et al. (2011)	USA	11	48	34/48 (71)	18/48 (38)	19/48 (40)
De Leeuw et al. (2013)	Belgium	n/a	47	19/47 (40)	13/47 (28)	n/a
Present study (2020)	Germany	10	46	46/46 (100)	15/46 (33)	19/46 (41)

^1^Frequently, no differentiation is made between the terms “suspect” and “perpetrator.” Instead, the term “perpetrator” is generally used in most studies, regardless of whether a confession or conviction is present

n/a = not applicable

*n* = number of cases

% = percent

The present study revealed that most criminal proceedings commenced with a complaint by the treating hospital (62%). In a US-American study by Ricci et al. [18], the authors reported that child protective services (CPS) were called in all cases (100%) by the hospitals. This observation may be plausibly explained by the fact that the treating hospitals are usually the first institutions where the injury patterns lead to the suspicion of child abuse, especially because multi-disciplinary diagnostic measures are crucial for the diagnosis of SBS/AHT. Furthermore, it was striking that, both in the study by Ricci et al. [18] and the present study, primary care pediatricians, who see the children during outpatient presentations, did not occur as complainants. This may be due to the high severity of the injuries prompting caregivers to go directly to a hospital. Additionally, primary care pediatricians probably tend to transfer children with suspected SBS/AHT to inpatient hospital care in order to confirm the diagnosis of child abuse and, thereby, avoiding the potentially unpleasant situation of reporting a criminal offense.

In the present study, criminal proceedings were found to be closed (without judgment) in 50% of the cases. In a similar study by Clauß et al. [26], investigating the consequences of criminal proceedings in cases of child abuse at one German university hospital, 33% (7 out of 21 cases) were dropped by the prosecution before starting a trial. “Lack of sufficient suspicion” was identified as the main reason for this observation in both studies. A very likely explanation may be the lack of the provability of the offense without confession by perpetrators or testimonies by independent witnesses. In addition, one of the main problems in the legal assessment of child abuse is the assignment of the perpetration to a specific perpetrator, i.e., the offense cannot clearly be ascribed to one offender. Usually, the perpetrator is alone with the child and belongs to the close family environment, while the young victims are often incapable of reporting on incidents. Finally, those situations then lead to the closing of the criminal proceedings based on the general legal principle “in dubio pro reo” ([when] in doubt, for the accused) according to German criminal law. Likewise, our findings of 41% of convicts in 46 SBS/AHT cases match with those available in the English-speaking literature [17–20], reporting rates of convictions ranging from 21 [17] to 47% [18] (Table 4).

The present study contains two criminal proceedings which were closed without judgment despite the presence of a confession to violent shaking by a perpetrator. In one case, the court decided that there was nonetheless no sufficient suspicion of the offense because the accused was subjected to pressure by in-laws. In the other case, the presence of a so-called brief resolved unexplained event (BRUE) prior to the violent shaking of the baby could not be excluded by the court. Therefore, the court interpreted the violent shaking as a plausible panic reaction due to the BRUE while attempting to fix a respiratory arrest of the baby.

According to a clinical practice guideline from the American Academy of Pediatrics (AAP) published by Tieder et al. [21], the term BRUE (formerly known as apparent life-threatening event, ALTE) is defined as “an event occurring in an infant younger than 1 year when the observer reports a sudden, brief, and now resolved episode of ≥ 1 of the following: (1) cyanosis or pallor; (2) absent, decreased, or irregular breathing; (3) marked change in tone (hyper- or hypotonia); and (4) altered level of responsiveness. Moreover, clinicians should diagnose a BRUE only when there is no explanation for a qualifying event after conducting an appropriate history and physical examination” (according to Tables 2 and 3 of that paper). The AAP also stated as early as in 2001 that “the act of shaking leading to shaken baby syndrome is so violent that individuals observing it would recognize it as dangerous and likely to kill the child” [27]. Therefore, from a medico-legal point of view, it still remains inadequate and implausible to conduct an apparent life-threating action in order to save a baby’s life.

When comparing different studies on AHT perpetrators, the question arises of who actually is a perpetrator. Starling et al. [17] grouped so-called perpetrators by their level of certainty in a descending order: first, caretakers who admitted injuring the child; second, persons convicted in a court of law; third, persons charged for the crime, but not convicted at the time of hospital discharge or loss of follow up; and fourth, caretakers who gave the police or medical evaluators a discrepant history which did not account for the injuries. Similarly, we decided to sub-divide defendants into suspects, convicts, and confessed perpetrators, thereby warranting an ascending level of certainty as to the perpetration.

Regarding the distribution of sexes among the suspects, convicts, and confessed perpetrators of our study, considerably more male than female perpetrators were found. As early as in the seminal article by Starling et al. from 1995 [17], male perpetrators outnumbered females (2.2:1), with fathers, step-fathers, and mothers’ boyfriends committing over 60% of the crimes. Table 5 shows a synopsis on the 3 most frequent suspects identified in studies on criminal proceedings concerning SBS/AHT cases. With exception of the study by Esernio-Jenssen et al. [20], who found mothers on rank no. 1, the fathers of the children were found as primary group of suspects in all studies (between 37 and 60%), including the present study (50%). Accordingly, Schnitzer and Ewigman [16] stated that mostly the parents themselves were identified as suspects and confessed perpetrators, however, not the new life partners of the parents. In our study, all perpetrators and 99% of the suspects are part of the close family environment of the injured children. While in 1995 Starling et al. [17] emphasized that especially female babysitters were identified as a large and previously unrecognized group of perpetrators (rank no. 3, 17.3%), the present study revealed only one suspect (1%) who had no familial relationship to the victim and was most likely the babysitter. In contrast to our findings and those of more recent literature, the perpetrators in the early study by Caffey from 1974 were mainly female [28], probably due to a change in the care situation that has occurred since then.

**Table 5:** Synopsis on the 3 most frequent suspects identified in studies on criminal proceedings concerning SBS/AHT cases (independent of confession or not)

	Rank 1	Rank 2	Rank 3
Starling et al. (1995)	Father (37%)	Boyfriend (21%)	Female babysitter (17%)
Ricci et al. (2003)	Father (66%)	Babysitter (13%)	Stepfather/boyfriend/mother (7% each)
Starling et al. (2004)	Father (45%)	Babysitter (18%)	Boyfriend (17%)
Kelly et al. (2009)	Father (60%)	Mother (25%)	Stepfather (10%)
Adamsbaum et al. (2010)	Father/stepfather^1^ (45%)	Mother (27%)	Child minder (21%)
Esernio-Jenssen et al. (2011)	Mother (29%)	Father (26%)	Boyfriend (21%)
Present study	Father (50%)	Mother (38%)	Boyfriend (3%)

^1^No distinction made

The age in the case group of the suspects ranges from 15 to 60 years, in the case group of the convicts from 18 to 39 years, and in confessed perpetrators from 18 to 30 years. The median ages in these groups show that the suspects, convicts, and especially confessed perpetrators were mostly young people. Young age and young maternal age in particular (approx. 18–26 years) is described as a parental risk factor for inflicted traumatic brain injury in young children [1].

Confessions by perpetrators remain a rare and indispensable source of information on the nature of SBS/AHT, even though their value is controversially discussed. For example, De Leeuw et al. stated that perpetrators who do confess often confess what the least socially reprehensible act is or provide the sort of confession that they expect to lead to the least criminal punishment [25]. Likewise, even false confessions may occur, for instance, in order to protect another person. A more detailed analysis of the content of the confessions, e.g., with respect to time-related or biomechanical aspects, or a comparison and matching with medical findings of the victims was not part of the present study as it focusses on the course and the outcome of the criminal proceedings. However, it was striking that the confessions were not very detailed in most cases. Basically, statements of violent shaking appeared to contain only a minimum of details necessary for being classifiable by the criminal court as a confession. This seemed not only to be due to restraint by the perpetrators but also to missing questions by the investigating authorities. So, if the suspect is willing to testify, it is recommended to ask for the smallest details of the act of violent shaking itself as well as for the detailed circumstances. This should be done in a standardized way, preferably as early as during the police investigation, e.g., by means of using a comprehensive questionnaire with a complete compilation of prefabricated questions from the field of SBS/AHT. This could create biomechanical evidence for the process of shaking and for the question of whether a symptom-free interval exists or not.

Apart from the two cases in which “calming” was additionally mentioned, the only reason for the violent shaking of the child stated by the confessed perpetrators is “excessive demand.” This observation perfectly accords with several previous studies where crying is discussed as a typical trigger for violent shaking [13–15, 24, 29]. That may be due to the frequently young age of the perpetrators including low frustration tolerance and impulse control, the socioeconomic status of the families concerned, and the index child being the first child of that family [1, 15].

Inherently, the present study contains several limitations. One major limitation is the retrospective study design. Thus, despite a 10-year period and a multi-center approach comprising data of three large university hospitals, only 46 out of 72 cases could finally be analyzed. A number of older case files had already been destroyed and were therefore unavailable. Another major limitation has to be seen in a selection bias as only children living at the time of the examination were included. However, it was a deliberate decision to waive primary autopsy cases of SBS/AHT for the present study. On the one hand, detailed clinical diagnostics is frequently not performed before the death of a child occurring suddenly, thus impeding an adequate comparability of mere autopsy and clinical cases. On the other hand, the study cohort should intentionally comprise cases from the clinical routine, i.e., cases with the typical SBS/AHT findings that pediatricians, neurosurgeons, ophthalmologists, radiologists, or clinical forensic physicians are frequently confronted with [8, 11, 12, 27, 30–39]. Hence, the present study cohort comprises cases with non-fatal outcome except for three. Concerning the case files available, the heterogeneity of the documentation, including the very different level of detail, considerably affected the acquisition and analysis of the data, representing another inevitable limitation of the study.

## Conclusions

1. The results of the present study are comparable with those of previous studies from other countries and, thereby, from other legal systems. Inherently, the direct comparability of legal systems from different countries is limited.
2. The medico-legal proof of the offense, i.e., the diagnosis of SBS/AHT, has never been at issue in the criminal proceedings investigated in the present study. Thus, it can be assumed that the current medico-legal procedures of securing evidence in cases of SBS/AHT may be sufficient to facilitate the court’s judgments in many cases. However, the Achilles heel of medico-legal investigations is the assignment of the perpetration to a specific perpetrator, which led to discontinuance of criminal proceedings in a considerable number of cases. Therefore, novel and more exact methods of forensic age estimation of the victim’s injuries would be desirable.
3. The present data corroborate the well-known insight that prevention programs have to focus on the close family environment, particularly on overstrained fathers who have repeatedly been identified as the most frequent perpetrators causing SBS/AHT.
4. Hospitals still play the major role in recognizing the victims of SBS/AHT. However, since, at this point, the concerned children have already sustained the dangerous assault, it would be desirable to sensitize primary care pediatricians and other early contact persons of potential victims in order to prevent more SBS/AHT cases in the future.

## References

[1] Keenan HT, Runyan DK, Marshall SW, Nocera MA, Merten DF, Sinal SH (2003). A population-based study of inflicted traumatic brain injury in young children. JAMA.

[2] Eisele JA, Kegler SR, Trent RB, Coronado VG (2006). Nonfatal traumatic brain injury-related hospitalization in very young children - 15 states, 1999. J Head Trauma Rehabil.

[3] Barlow KM, Thomson E, Johnson D, Minns RA (2005). Late neurologic and cognitive sequelae of inflicted traumatic brain injury in infancy. Pediatrics.

[4] Ellingson KD, Leventhal JM, Weiss HB (2008). Using hospital discharge data to track inflicted traumatic brain injury. Am J Prev Med.

[5] Jayawant S, Rawlinson A, Gibbon F, Price J, Schulte J, Sharples P, Sibert JR, Kemp AM (1998). Subdural haemorrhages in infants: population based study. BMJ.

[6] Niederkrotenthaler T, Xu L, Parks SE, Sugerman DE (2013). Descriptive factors of abusive head trauma in young children--United States, 2000–2009. Child Abuse Negl.

[7] Talvik I, Metsvaht T, Leito K, Põder H, Kool P, Väli M, Lintrop M, Kolk A, Talvic T (2006). Inflicted traumatic brain injury (ITBI) or shaken baby syndrome (SBS) in Estonia. Acta Paediatr.

[8] Matschke J, Herrmann B, Sperhake J, Körber F, Bajanowski T, Glatzel M (2009). Shaken baby syndrome: a common variant of non-accidental head injury in infants. Dtsch Arztebl Int.

[9] Christian CM, Block R, Committee on Child Abuse and Neglect (2009). Abusive head trauma in infant and children. Paediatrics.

[10] Sperhake J, Herrmann B (2008). Schütteltrauma (nichtakzidentelle Kopfverletzung). Aktuelle Kontroversen Rechtsmedizin.

[11] Sperhake J, Matschke J (2014). “Shaken baby syndrome” and forensic pathology. Forensic Sci Med Pathol.

[12] Choudhary AK, Servaes S, Slovis TL, Palusci VJ, Hedlund GL, Narang SK, Moreno JA, Dias MS, Christian CW, Nelson MD, Silvera VM, Palasis S, Raissaki M, Rossi A, Offiah AC (2018). Consensus statement on abusive head trauma in infants and young children. Pediatr Radiol.

[13] Barr RG, Trent RB, Cross J (2006). Age-related incidence curve of hospitalized shaken baby syndrome cases: convergent evidence for crying as a trigger to shaking. Child Abuse Negl.

[14] Barr RG, Barr M, Rajabali F, Humphreys C, Pike I, Brant R, Hlady J, Colbourne M, Fujiwara T, Singhal A (2018). Eight-year outcome of implementation of abusive head trauma prevention. Child Abuse Negl.

[15] Talvik I, Alexander RC, Talvik T (2008). Shaken baby syndrome and a baby’s cry. Acta Paediatr.

[16] Schnitzer PG, Ewigman BG (2005). Child deaths resulting from inflicted injuries: household risk factors and perpetrator characteristics. Pediatrics.

[17] Starling SP, Holden JR, Jenny C (1995). Abusive head trauma: the relationship of perpetrators to their victims. Pediatrics.

[18] Ricci L, Giantris A, Merriam P, Hodge S, Doyle T (2003). Abusive head trauma in Maine infants: medical, child protective, and law enforcement analysis. Child Abuse Negl.

[19] Kelly P, MacCormick J, Strange R (2009). Non-accidental head injury in New Zealand: the outcome of referral to statutory authorities. Child Abuse Negl.

[20] Esernio-Jenssen D, Tai J, Kodsi S (2011). Abusive head trauma in children: a comparison of male and female perpetrators. Pediatrics.

[21] Tieder JS, Bonkowsky JL, Etzel RA, Franklin WH, Gremse DA, Herman B, Katz ES, Krilov LR, Merritt JL, Norlin C, Percelay J, Sapién RE, Shiffman RN, Smith MB (2016). Brief resolved unexplained events (formerly apparent life-threatening events) and evaluation of lower-risk infants: executive summary. Pediatrics.

[22] Starling SP, Patel S, Burke BL, Sirotnak AP, Stronks S, Rosquist P (2004). Analysis of perpetrator admissions to inflicted traumatic brain injury in children. Arch Pediatr Adolesc Med.

[23] Biron D, Shelton D (2005). Perpetrator accounts in infant abusive head trauma brought about by a shaking event. Child Abuse Negl.

[24] Adamsbaum C, Grabar S, Mejean N, Rey-Salmon C (2010). Abusive head trauma: judicial admissions highlight violent and repetitive shaking. Pediatrics.

[25] De Leeuw M, Beuls M, Parizel P, Jorens P, Jacobs W (2013). Confessed abusive blunt head trauma. Am J Forensic Med Pathol.

[26] Clauß D, Richter C, Klohs G, Heide S (2013). Legal consequences in cases of child abuse. Klin Padiatr.

[27] American Academy of Pediatrics: Committee on Child Abuse and Neglect (AAP) (2001). Shaken baby syndrome: rotational cranial injuries-technical report. Pediatrics.

[28] Caffey J (1974). The whiplash shaken infants syndrome: manual shaking by the extremities with whiplash-induced intracranial and intraocular bleedings, linked with residual permanent brain damage and mental retardation. Pediatrics.

[29] (2001) Joint statement on shaken baby syndrome. Paediatr Child Health 6:663–667 10.1093/pch/6.9.663 PMC2805972 20084140

[30] Case ME, Graham MA, Handy TC, Jentzen JM, Monteleone JA, National Association of Medical Examiners Ad Hoc Committee on Shaken Baby Syndrome (2001). Position paper on fatal abusive head injuries in infants and young children. Am J Forensic Med Pathol.

[31] Levin AV (2010). Retinal hemorrhage in abusive head trauma. Pediatrics.

[32] Hahnemann ML, Kinner S, Schweiger B, Bajanowski T, Karger B, Pfeiffer H, Wittschieber D (2015). Imaging of bridging vein thrombosis in infants with abusive head trauma: the “tadpole sign”. Eur Radiol.

[33] Homa A, Nentwich M (2018). Retinal hemorrhages in shaken baby syndrome. Differential diagnostic aspects. Rechtsmedizin.

[34] Wittschieber D, Kinner S, Pfeiffer H, Karger B, Hahnemann ML (2018). Forensic aspects of imaging procedures in shaken baby syndrome. Methodology, findings, differential diagnoses. Rechtsmedizin.

[35] Wittschieber D, Karger B, Pfeiffer H, Hahnemann ML (2019). Understanding subdural collections in pediatric abusive head trauma. AJNR Am J Neuroradiol.

[36] Orman G, Kralik SF, Meoded A, Desai N, Risen S, Huisman TAGM (2020). MRI findings in pediatric abusive head trauma: a review. J Neuroimaging.

[37] Al-Saadoon M, Elnour IB, Ganesh A (2011). Shaken baby syndrome as a form of abusive head trauma. Sultan Qaboos Univ Med J.

[38] Feld K, Banaschak S, Remschmidt H, Rothschild MA (2018). Shaken baby syndrome - what convicted perpetrators report. Rechtsmedizin.

[39] Leestma JE (2005). Case analysis of brain-injured admittedly shaken infants: 54 cases, 1969-2001. Am J Forensic Med Pathol.

